# A geotagged image dataset with compass directions for studying the drivers of farmland abandonment

**DOI:** 10.1016/j.dib.2020.106340

**Published:** 2020-09-23

**Authors:** B. Zaragozí, S. Trilles, D. Carrion, M.Y. Pérez-Albert

**Affiliations:** a Departament de Geografia, Universitat Rovira i Virgili, C/Joanot Martorell 15, Vilaseca, 43480, Spain; b Institute of New Imaging Technologies, Universitat Jaume I, Av. Vicente Sos Baynat s/n, Castelló de la Plana, 12071, Spain; c Geodesy and Geomatics Section, DICA, Politecnico di Milano, Piazza Leonardo Da Vinci 32, Milano, 20133, Italy

**Keywords:** Geotagged photos, Exif, GIS, Viewshed

## Abstract

In this work, we present a dataset containing a collection of pictures taken during the fieldwork of a farmland abandonment study. Data was taken in 2010 with a compact camera that incorporates GPS and a digital compass sensor. The photographs were taken as part of a GIS database. Using their Exif metadata, we created a layer of geographic fields of view (geoFOVs) that can be used to perform specific spatial queries. The dataset contains 2,235 pictures and GIS layers of geoFOVs contextualising the agricultural plots being photographed. The dataset is hosted in a Zenodo dataset repository.

## Specifications Table

**Table:** 

Subject	Geography
Specific subject area	Geographical information systems
Type of data	Pictures and a GIS layers
How data were acquired	Survey and feature extraction
Data format	Raw, filtered, and analysed
Parameters for data collection	Pictures were taken from public roads showing structurant elements and agricultural practices.
Description of data collection	The pictures are taken to show structurant elements and agricultural practices that remain in this region.
Data source location	Marina Baja, Alicante, Spain (region of interest in the GIS file)
Data accessibility	Zaragozí, Benito (2020), “A geotagged image dataset with compass directions for studying the drivers of farmland abandonment”, Zenodo, V1, doi: 10.5281/zenodo.3733436 Usage rights: Creative Commons Attribution 4.0 International license (CC BY 4.0)
Related research article	Zaragozí, B., Rabasa, A., Rodríguez-Sala, J. J., Navarro, J. T., Belda, A., Ramón, A., et al. (2012). Modelling farmland abandonment: A study combining GIS and data mining techniques. Agriculture, Ecosystems and Environment, 155, 124–132. DOI: 10.1016/j.agee.2012.03.019

## Value of the Data

• The data can be used for testing new GIS methods for creating better viewsheds and 3D models from photographs.
• This data could be used to perform a study on the evolution of this area. Repeating these pictures from the same vantage point would be easy thanks to the stored attributes (repeat photography or rephotography).
• The dataset can be useful for research in feature extraction, semantic enrichment, and query service for geotagged photographs (e.g. estimating sun position).

## 1 Data Description

**Fig 1:**
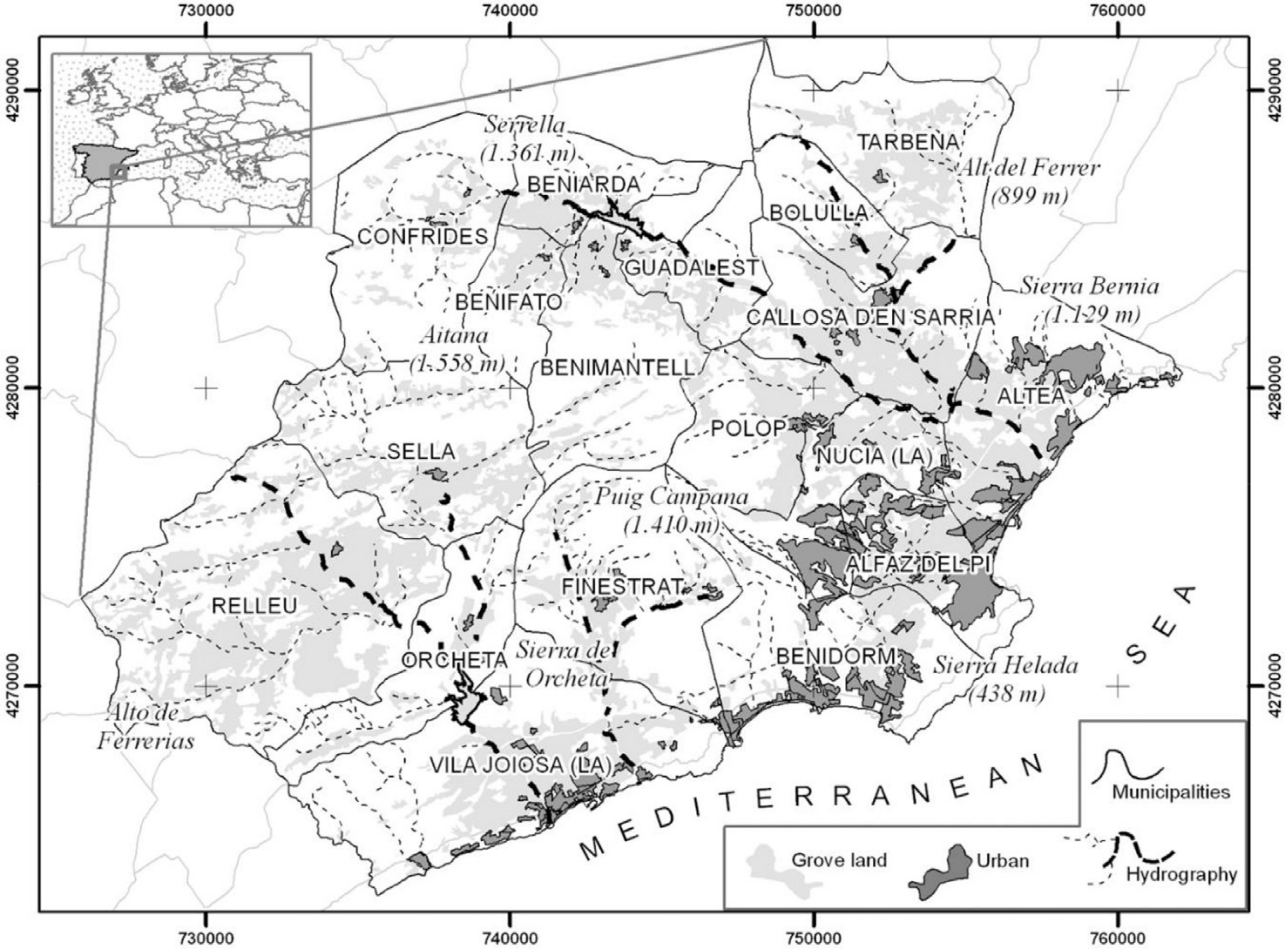
Map of the study area (Zaragozí et al., 2012).

This article presents a dataset generated during farmland abandonment research [1]. The study area is the Marina Baja region in the south-east of Spain. This is a typical Mediterranean region in which there is intense competition between land uses, especially between urban-tourism and agroforestry uses. The location of the studied region is shown in Fig. 1. The dataset is divided into two parts: the first part contains 2,235 fieldwork pictures taken in 2010. The images were captured with a Sony DSC-HX5V compact camera. This device retrieves GPS coordinates and compass orientation for each captured image. The images are stored in the well-known Joint Photographic Experts Group (JPEG) standard format, with an Exif header containing geographic and non-geographic metadata [2]. The metadata attributes stored in those images are summarised in Table 1. The second part of the dataset contains vector layers in the GeoJSON, which is an Internet Engineering Task Force (IETF) standard format (RFC 7946). This dataset contains geometries representing the geographical field of view (FOV) of each photograph. According to the GeoJSON format, these geometries are stored using a geographic coordinate reference system (World Geodetic System 1984) and units of decimal degrees.

**Table 1:** Geographic tags in the Exif 2.2 standard, highlighting those that can be found in the images in this dataset.

Captured by Sony DSC-HX5V	Not captured
	
• GPS tag version *[GPSVersionID]* • North or south latitude *[GPSLatitudeRef]* • Latitude *[GPSLatitude]* • East or west longitude *[GPSLongitudeRef]* • Longitude *[GPSLongitude]* • Altitude reference *[GPSAltitudeRef]* • Altitude *[GPSAltitude]* • GPS time (atomic clock) *[GPSTimeStamp]* • GPS receiver status *[GPSStatus]* • GPS measurement mode *[GPSMeasureMode]* • Speed unit *[GPSSpeedRef]* • Speed of GPS receiver *[GPSSpeed]* • Reference for direction of movement *[GPSTrackRef]* Direction of movement *[GPSTrack]* • Reference for direction of image *[GPSImgDirectionRef]* • Direction of image *[GPSImgDirection]* • Geodetic survey data used *[GPSMapDatum]* • GPS date *[GPSDateStamp]* • GPS differential correction *[GPSDifferential]*	• GPS satellites used for measurement *[GPSSatellites]* • Measurement precision *[GPSDOP]* • Reference for latitude of destination *[GPSDestLatitudeRef]* • Latitude of destination *[GPSDestLatitude]* • Reference for longitude of destination *[GPSDestLongitudeRef]* • Longitude of destination *[GPSDestLongitude]* • Reference for bearing of destination *[GPSDestBearingRef]* • Bearing of destination *[GPSDestBearing]* • Reference for distance to destination *[GPSDestDistanceRef]* • Distance to destination *[GPSDestDistance]* • Name of GPS processing method *[GPSProcessingMethod]* • Name of GPS area *[GPSAreaInformation]*

In the last two decades, the number of captured digital photographs has significantly increased, which opens up many opportunities for research if this information is managed correctly. However, it is not just about the images. The metadata that accompanies these images and the parameters that can be derived from the images themselves are also of great value. In the dataset shared here some of these metadata are of particular interest due to the detailed geographic context. In particular, the Exif standard includes a specific section on geographical labels (see Table 1), and other more general labels, that may be of interest to describe the conditions in which each photograph was taken.

From a technical point of view, such metadata can be exploited through powerful databases and semantic query systems, but it is also possible to take advantage of certain parameters to delve into the geographical context in which each photograph was captured [3,4]. For example, it is important to know the position of the photographic camera when validating metadata [5]. In certain circumstances, it is possible to derive certain characteristics from the information contained in the photograph itself, such as the relative position of the sun with respect to the camera, or the approximate configuration of the camera at the time of capture [6,7]. To validate this type of calculation, other authors have taken advantage of data sources such as aerial photographs, Street View, or 3D models [8,9]. In these types of analyses, it would be interesting to have a metadata registry that facilitates the validation of the calculations in a more direct way. The geotagged image dataset provided here could be useful in testing and validating new feature extraction algorithms.

More specifically, the value of geo-referenced images for landscape and rural studies has been addressed by different authors [10,11]. In the European context, the use of geotagged photographs has been recommended, even in the management of CAP (Common Agricultural Policy) subsidies, since these images provide a level of information that remote images cannot reach, even providing evidence of when mapping parcels need to be updated [12]. In this context of landscape studies, this dataset provides detailed information for validating different assumptions about the drivers of abandonment of a Mediterranean area [1,^13^,14].

## 2 Experimental Design, Materials and Methods

### 2.1 Study area description

The Marina Baja is a Spanish region located at the south-east of the Iberian Peninsula, some 50 km north-east of the city of Alicante and 120 km south of Valencia. It is a small region (only 578.5 km^2^), with 40 km of coastline (Fig. 1). It contains 18 municipalities, including Benidorm, which is the most populated city with 67.558 inhabitants and a well-known tourist destination in Spain. This small area is diverse, and despite its distance from the sea, it contains several relatively high peaks (the highest being 1,129 meters in the Sierra de Bèrnia and the 1,558 metres in the Sierra de Aitana). Precipitation values range from north to south between 826 mm/year in Tarbena and 280 mm/year in Benidorm. The heights of the mountains and the distance to the sea explain the small differences of temperatures across the area. Over the last two decades, there has been a profound change in land use and land cover. According to the Spanish Agricultural Census, between 1999 and 2009, up to 50% of the farmland in this region was abandoned or changed into other activities [15,16]. This process differed in areas where irrigated agriculture was still productive during the last years of the 20th century. The Guadalest-Algar river area was home for decades to productive agriculture based on fruit and citrus trees. Since the turn of the century, due to the lower prices of agricultural products and the ageing of the population — added to the slowdown in the property market — changes in land use tend to the abandonment of the least productive plots.

### 2.2 Fieldwork, picture collection, and analytical procedures

The photographs were taken with a Sony DSC-HX5V camera with a GPS receiver and digital compass allowing for the acquisition of the geographic coordinates and the picture azimuth. Fieldwork was developed during 65 working days. Between January and May 2009, all the municipalities of the region were visited, in attempts to reach those plots that seemed abandoned in aerial images. More than 17,000 photographs were taken, most of them in the vicinity of accessible roads. However, many other images were taken from natural viewpoints, or on the many private paths that are found in the mountain municipalities. From this raw dataset, we selected 2,235 photographs of the north of the Marina Baja that enable the plots to be classified according to the following situations:

• Absence of traditional agricultural practices.
• State of conservation of the stone walls.
• Presence of signs of forest recolonisation, or totally recolonised agricultural land.
• Evidence of the effects of abandonment, such as erosion or forest fires.
• Very heterogeneous plant sizes.
• No use of the land is appreciated on the plot, but in the official database recent agricultural use is indicated.
• There are structural elements that indicate recent investment.

**Fig 2:**
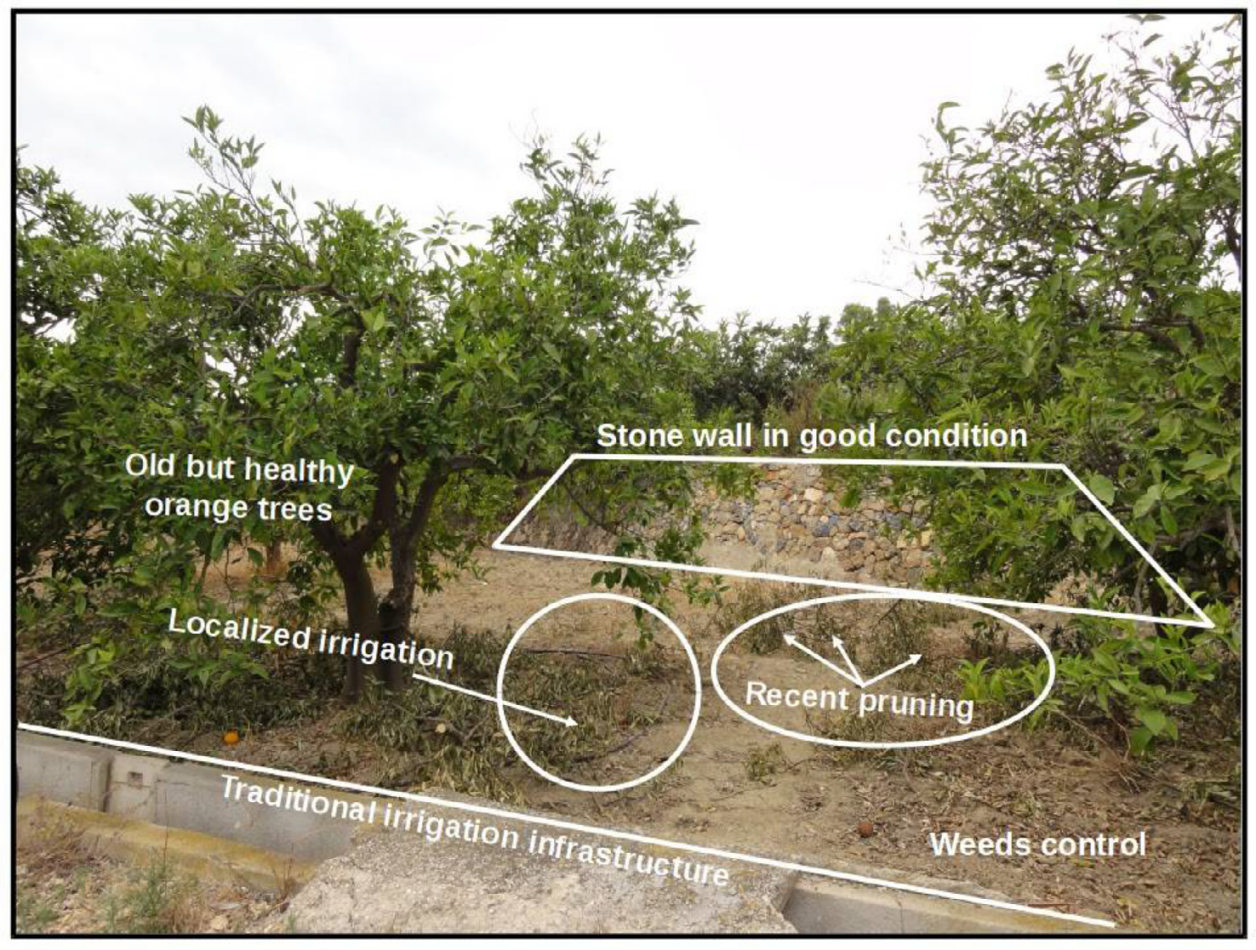
Example of an agricultural plot of irrigated land in good condition (image ‘DSC00088.JPG’ from the dataset).

In Fig. 2 there is an example of an image that collects all the information of interest of a plot in irrigated areas. In this image, a plot with quite old orange trees can be seen, where the stonewalls are well preserved, the irrigation system has been modernised, pruning is still being practised, and weeds were recently eliminated.

**Fig 3:**
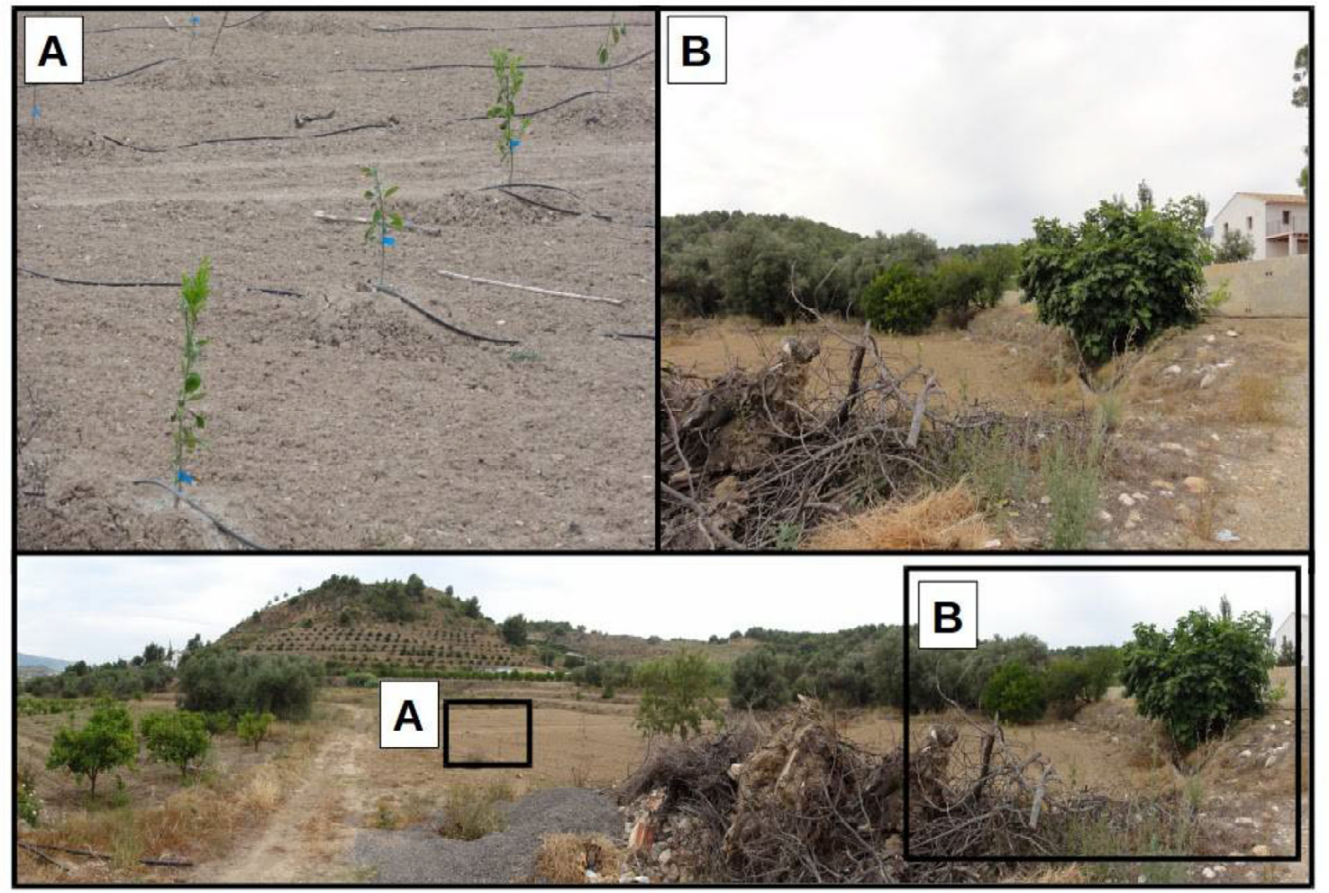
Example of a panorama where details of a new citrus plot are extracted (image ‘DSC00071.JPG’ from the dataset).

In the picture dataset, many panoramic images provide context data on more than one plot. For example, in Fig. 3, we can see an individual plot where two different development stages coexist. In this figure, we can see how the traditional rain-fed agriculture (3.B) is being replaced by a more technical approach (3.A).

### 2.3 Building geoFOVs

The camera device stored all the necessary metadata to estimate the image viewshed. As mentioned above, the position is stored in the Exif metadata, together with other details, such as the parameters of the image acquisition, date, time, among other standard attributes. From these metadata, a horizontal field of view (HFOV) can be built following the steps detailed in this section. Once the geometric representations of HFOV are created, they enable different types of spatial queries to be carried out (e.g. applying spatial filters, or joining the information extracted from the photographs to any GIS layer).

**Fig 4:**
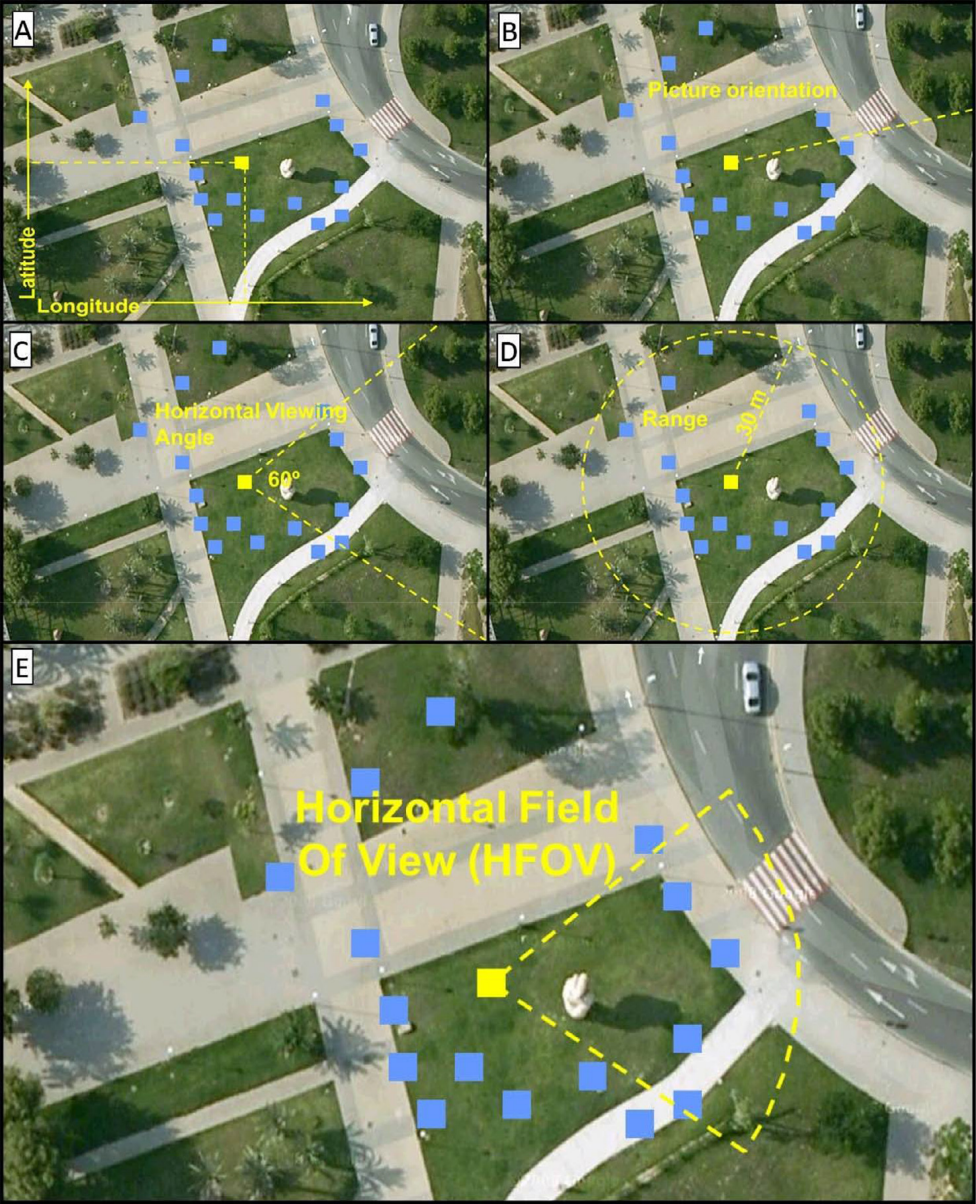
Visualisation of the steps for drawing a GIS FOV. A) latitude and longitude; B) image direction; C) calculating HFOV from camera sensor and focal length; D) Buffer distance of fieldwork detail; E) Geometry estimating the potential viewshed of the image (GeoFOV).

For the sake of clarity, we explain these steps using a small test set of pictures taken around a sculpture of a hand. In Fig. 4 we provide a visual example to understand how the viewsheds of the images were estimated. This figure shows the basic steps of the algorithm to construct an area of visibility (or geoFOV) in two dimensions. Sub-Fig. 4.A shows that it is necessary to know the geographical coordinates from which the photograph was taken, obtained from a GPS device integrated in the camera. Sub-Fig. 4.B points out that it is also necessary to know the orientation of the photograph, which is achieved thanks to the integrated digital compass. Sub-Fig. 4.C shows the result of calculating the horizontal field of view of the image, for which it is necessary to know details of the lens and focal length with which the image was captured. Fig. 4.D represents an arbitrary decision of the maximum distance for which the photograph is considered relevant. This distance is materialised by applying a buffer area that could vary, since the Sony DSC-HX5V camera focuses at infinity, but the result of the buffer could also be intersected with other SIG layers, or digital models of the terrain to obtain a more precise result. Finally, the last subfigure shows the result of intersecting the previous geometries, obtaining the shape that represents the horizontal field of view. In the following subsections, some code snippets and simple query examples show the great potential for these types of calculations in landscape change studies.

### 2.4 Read Exif metadata from images

To locate the picture over a map, it is necessary to know the position from which the image was taken, but also to draw the polygon corresponding to the scene appearing in the photo, i.e. its footprint. To do so, some acquisition parameters must be known, such as the azimuth of the camera when the image was taken, the focal length, and the depth of field. Exif metadata enables storing the necessary metadata to calculate geoFOV: GPSDestLongitude and GPSDestLatitude (Fig. 4.A), GPSImgDirection (Fig. 4.B), and the FocalLength, which is necessary for calculating the HFOV (Fig. 4.C). Finally, a buffer range is specified that adjusts the detail of the fieldwork (Fig. 4.D). It must be explained that there are different approaches for calculating the orientation of a picture. Some software platforms use the bearing of a GPS track (GPSDestBearing) when there is not a real measurement of the real camera orientation (GPSImgDirection).

### 2.5 Calculate the HFOV angle

**Fig 5:**
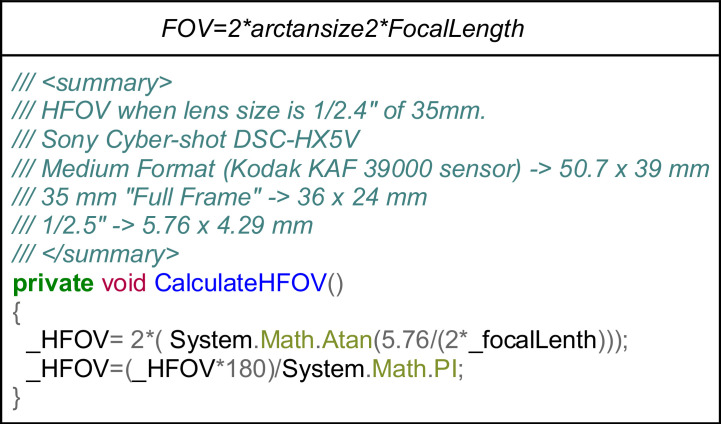
Calculation of the camera HFOV. Top) Equation relating the dimension of the sensor in millimetres with the focal length; Bottom) C-sharp code snippet considering a 5.76 mm horizontal sensor size.

Considering the geometry of the optical acquisition, the angle corresponding to the field of view (FOV) can be computed, as shown in Fig. 5. The Sony Cyber-shot DSC-HX5V technical specifications were used to estimate the sensor size. This information was not stored in the metadata, which would have been very useful.

**Fig 6:**
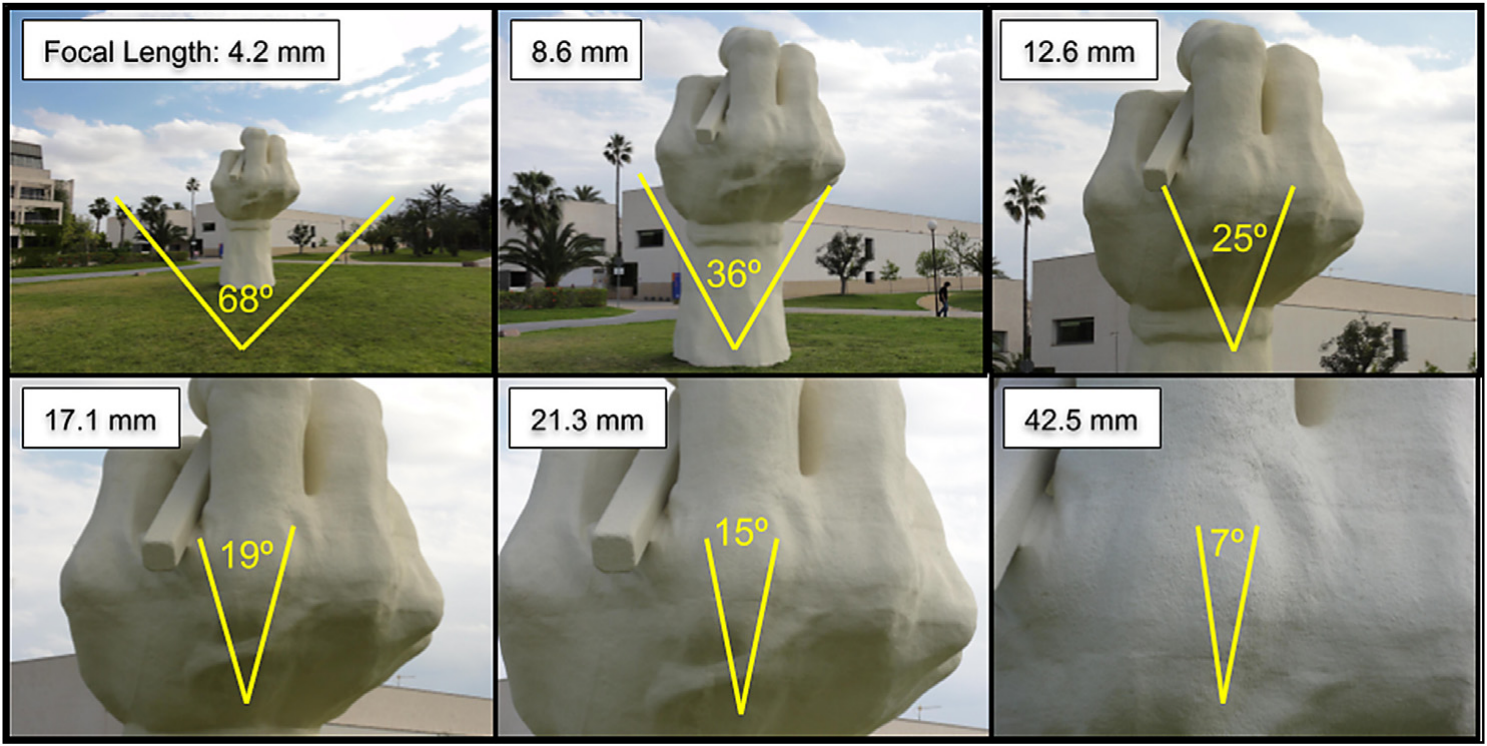
Focal length effect on the horizontal field of view. Six captures from the same point of view.

In this compact camera with 10x optical zoom, the focal length parameter is the only variable affecting the HFOV. In Fig. 6, there is an example of how the FocalLength affects HFOV when zooming in the scene.

### 2.6 Build the geometries

**Fig 7:**
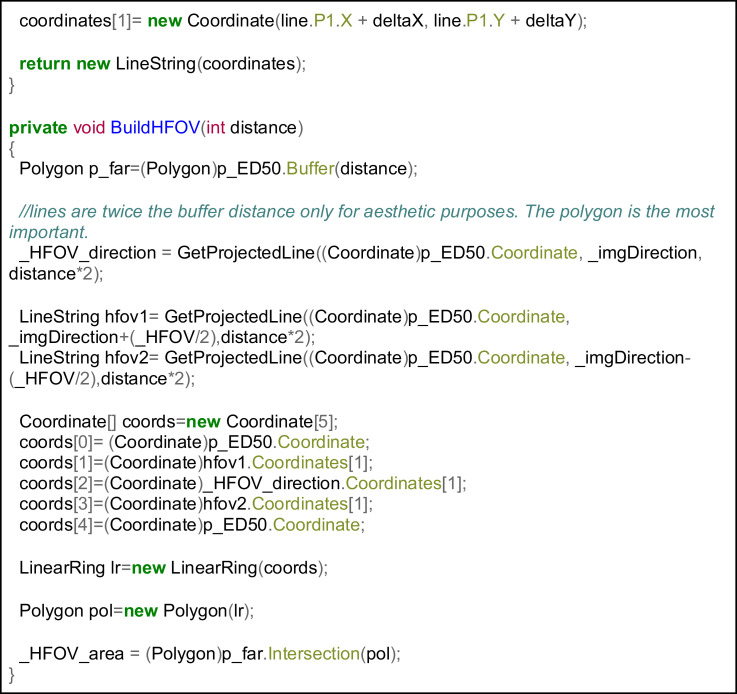
Focal length effect on the horizontal field of view.

**Fig 8:**
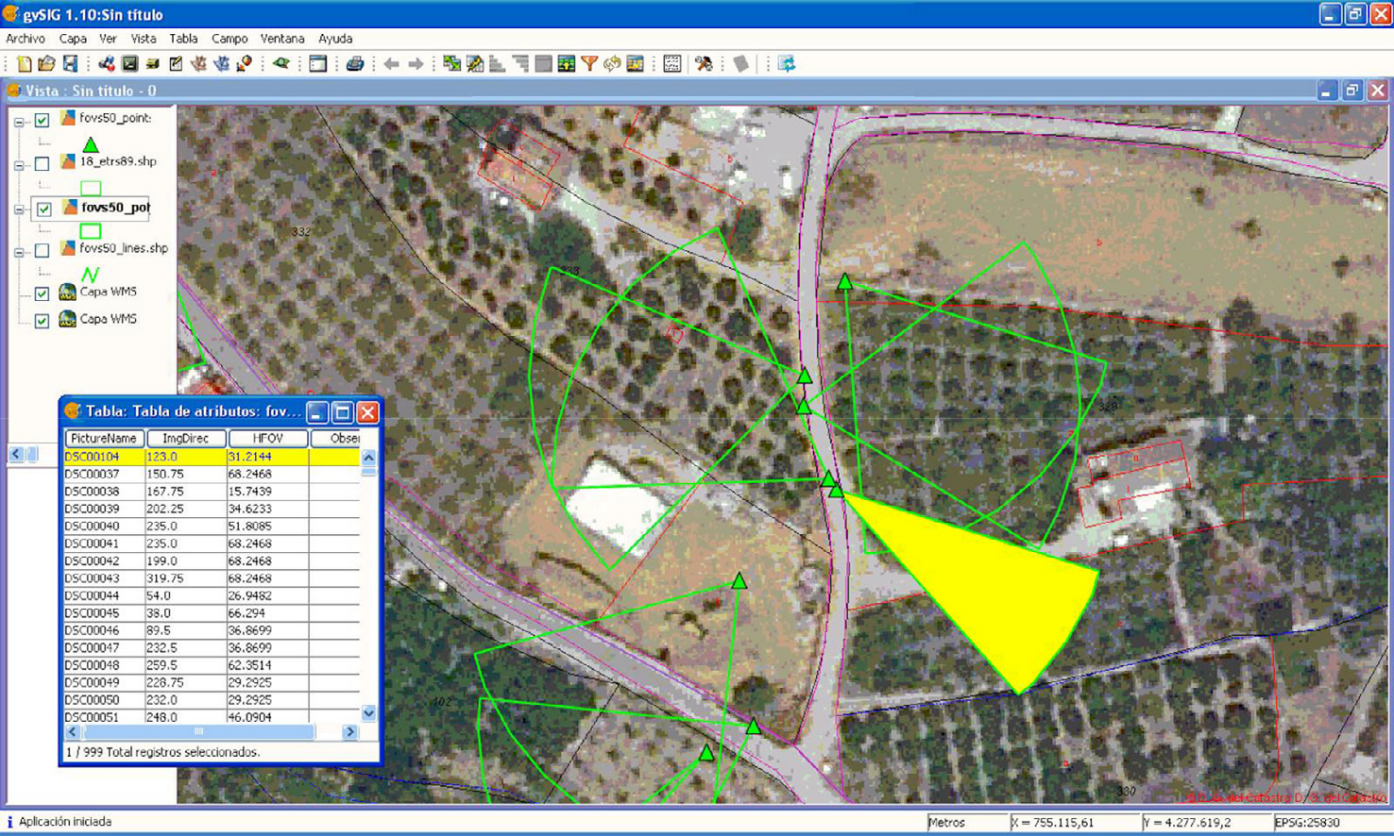
Example of a spatial query based on composite predicates. Retrieving pictures showing three elements: tree, road and sculpture. Only one image in the dataset satisfies those three requirements.

**Fig 9:**
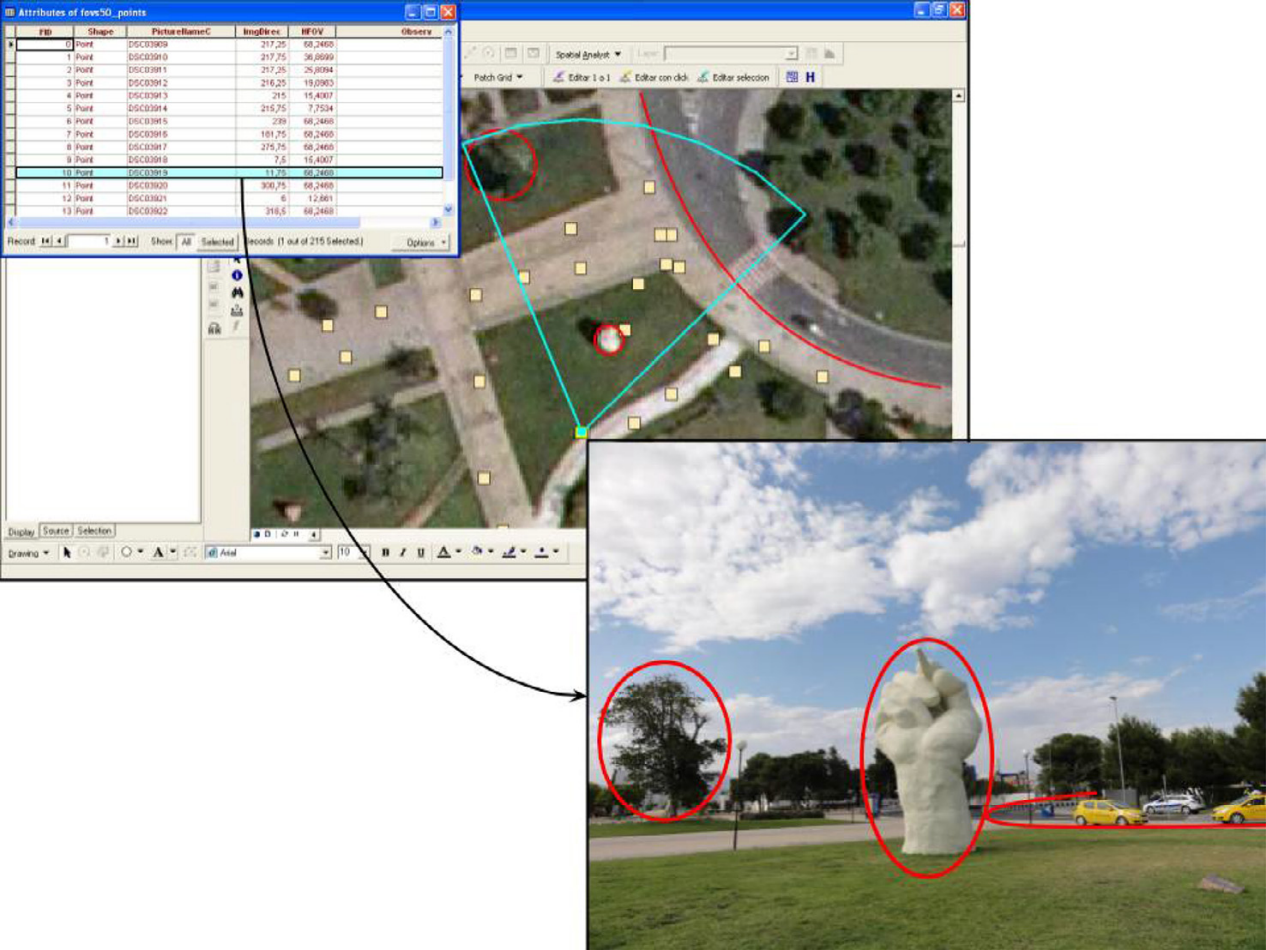
Using gvSIG for querying the image database. Select all the images showing a specific agricultural plot.

Following the steps detailed in Fig. 4, a distance of 50 meters was specified. The camera automatically focused to infinity but, considering the purpose of the images, it was decided that 50 meters was sufficient according to the expected observation detail (agricultural plot level). The code snippet in Fig. 7 shows how this buffer can be calculated. The HFOV was then used to create a triangle pointing to the camera location and, finally, this triangle is intersected with the buffer. Figs. 8 and 9 show examples of these calculations.

### 2.7 GeoFOV based GIS queries

Combining the photocollection and the computed geometries in a GIS database may have several potential applications. Using these geoFOVs, specific GIS queries could be performed. Fig. 8 shows the most basic approach for querying the provided dataset on farmland abandonment pictures. There are two basic queries to perform: (1) the direct query (what can be seen from a photograph?), and (2) the reverse query (which photographs show a certain point in a landscape?). Additionally, this type of query could even be performed through spatial SQL queries for answering more complex questions, or the geoFOVs could be calculated dynamically, specifying different buffer distances.

Finally, as an example of more complex queries, Fig. 9 shows a query filtering those pictures where three different elements appear, returning only one image that could be further analysed. Of course, the definition of the buffer distance and the elevations in the area are significant for this approach to succeed, but considering the parametrisation of the fieldwork (see Section 2.2), this was not a problem in the provided geotagged image dataset.

## CRediT authorship contribution statement

**B. Zaragozí:** Data curation, Formal analysis, Investigation, Methodology, Resources, Software, Validation, Visualization, Writing - original draft. **S. Trilles:** Investigation, Methodology, Resources, Software, Validation, Visualization, Writing - review & editing. **D. Carrion:** Formal analysis, Investigation, Resources, Supervision, Writing - review & editing. **M.Y. Pérez-Albert:** Formal analysis, Funding acquisition, Investigation, Project administration, Resources, Supervision, Writing - review & editing.

## Declaration of Competing Interest

The authors declare that they have no known competing financial interests or personal relationships which have, or could be perceived to have, influenced the work reported in this article.

## Appendix Supplementary materials

Image, application 1

## References

[1] Zaragozí B., Rabasa A., Rodríguez-Sala J.J., Navarro J.T., Belda A., Ramón A. (2012). Modelling farmland abandonment: a study combining GIS and data mining techniques. Agric. Ecosyst. Environ..

[2] Japan Electronics & Information Technology Industries Association (2002). Exchangeable Image File Format for Digital Still Cameras: EXIF Version 2.2 JEITA CP-3451. https://www.exif.org/Exif2-2.PDF.

[3] Ennis A., Nugent C., Morrow P., Chen L., Ioannidis G., Stan A., Rachev P. (2015). A geospatial semantic enrichment and query service for geotagged photographs. Sensors.

[4] Mousselly-Sergieh H., Watzinger D., Huber B., Döller M., Egyed-Zsigmond E., Kosch H. (2014). World-wide scale geotagged image dataset for automatic image annotation and reverse geotagging. Proceedings of the 5th ACM Multimedia Systems Conference.

[5] Tsai T.H., Jhou W.-C., Cheng W.-H., Hu M.-C., Shen I.-C., Lim T., Hua K.-L., Ghoneim A., Hossain M.A., Hidayati S.C. (2016). Photo sundial: estimating the time of capture in consumer photos. Neurocomputing.

[6] Wehrwein S., Bala K., Snavely N. (2015). Shadow detection and sun direction in photo collections. Proceedings of the International Conference on 3D Vision.

[7] Lalonde J.F., Narasimhan S.G., Efros A.A. (2010). What do the sun and the sky tell us about the camera?. Int. J. Comput. Vis..

[8] Park M., Luo J., Collins R.T., Liu Y. (2014). Estimating the camera direction of a geotagged image using reference images. Pattern Recognit..

[9] Yin J., Carswell J.D. (2013). Spatial search techniques for mobile 3D queries in sensor web environments. ISPRS Int. J. Geoinf..

[10] Yoshimura N., Hiura T. (2017). Demand and supply of cultural ecosystem services: use of geotagged photos to map the aesthetic value of landscapes in Hokkaido. Ecosyst. Serv..

[11] Foltête J.C., Ingensand J., Blanc N. (2020). Coupling crowd-sourced imagery and visibility modelling to identify landscape preferences at the panorama level. Landsc. Urban Plan..

[12] Sima A. (2017). Geotagged images for capturing additional evidence. Proceedings of the 23rd MARS Conference.

[13] Zaragozí B., Navarro J.T., Ramón Morte A., Rodríguez Sala J. (2011). A study of drivers for agricultural land abandonment using GIS and data mining techniques. Proceedings of the Eighth International Conference On Ecosystems and Sustainable Development (ECOSUD VIII).

[14] B. Zaragozí, J.J. Rodríguez, A. Rabasa, A. Ramon, J. Olcina, others, A data driven study of relationships between relief and farmland abandonment in a Mediterranean region, in: A. Marinov, University Politehnica of Bucharest, Romania; C.A. Brebbia (Eds.) Ecosystems and Sustainable Development IX, 175, pp. 219–230.

[15] INE, (1999). Censo Agrario de 1999—Base de Microdatos [Agricultural Census 1999—Microdata Base].

[16] INE, (2009). Censo Agrario de 2009—Base de Microdatos [Agricultural Census 2009—Microdata Base].

